# Uterine vascular resistance and other maternal factors associated with the risk of developing hypertension during pregnancy

**DOI:** 10.1590/1414-431X202010118

**Published:** 2020-11-18

**Authors:** L.A.B. Martins, E.C.A. Veiga, C.C.C. Ribeiro, V.M.F. Simões, V.C. Cardoso, H. Bettiol, M.A. Barbieri, R.C. Cavalli

**Affiliations:** 1 Faculdade de Ciências da Saúde de Barretos Dr. Paulo Prata, Barretos, SP, Brasil; 2 Departamento de Ginecologia e Obstetrícia, Faculdade de Medicina de Ribeirão Preto, Universidade de São Paulo, Ribeirão Preto, SP, Brasil; 3 Departamento de Odontologia II, Universidade Federal do Maranhão, São Luís, MA, Brasil; 4 Departamento de Saúde Pública, Universidade Federal do Maranhão, São Luís, MA, Brasil; 5 Departamento de Puericultura e Pediatria, Faculdade de Medicina de Ribeirão Preto, Universidade de São Paulo, Ribeirão Preto, SP, Brasil

**Keywords:** Gestational hypertension, Risk factors, Relative risk

## Abstract

Gestational hypertension and pre-eclampsia are important causes of perinatal morbidity. The objective of the present study was to determine the increase in relative risk for developing hypertensive disorders of pregnancy based on the evaluation of pregnant women between 20 and 25 weeks of gestation, and to correlate the findings at this period with the outcome of pregnancy. We conducted a prospective cohort study, with a convenience sample of 1417 patients evaluated at this gestational age, of which 1306 were contacted at childbirth. We detected an increased relative risk of 2.69 (95%CI: 1.86 to 3.89) associated with pulsatility index of the uterine arteries, a 2.8 increase (95%CI: 1.58 to 5.03) in relative risk attributed to maternal age above 35 years, a 1.68 increase (95%CI: 1.17 to 2.40) attributed to parity greater than or equal to 3, and a 5.35 increase (95%CI: 4.18 to 6.85) attributed to chronic hypertension and obesity, with a progressive increase in relative risk according to the degree of overweight, i.e., grades 1, 2, 3, and morbid obesity (2.58, 3.06, 5.84, and 7.28, respectively).

## Introduction

Arterial hypertension during pregnancy is an important cause of perinatal morbidity and mortality. Although its pathophysiology has not been fully clarified, its etiology is believed to be multifactorial, with an unlikely possibility that a single theory can explain all the cases observed (1). Endothelial factors linked to reduced uterine vascular resistance are strongly correlated with the development of gestational hypertension (2,3). With an incidence ranging from 4 to 10% of all pregnancies, hypertensive disorders continue to be the main cause of maternal-fetal morbidity and mortality (4).

Several studies have suggested that endothelial factors linked to reduced uterine vascular resistance are strongly correlated with the development of gestational arterial hypertension (GAH) (4,5). Some factors may be associated with pressure changes during pregnancy. Obese pregnant women have a higher incidence of gestational arterial hypertension compared to the normal population (6). Maternal age also influences the risk of developing hypertensive disorders during pregnancy, with an increase in the incidence of gestational hypertension and pre-eclampsia (PE) among pregnant women older than 35 years, and a more significant increase after 40 years of age (7). Parity may also be related to an increased risk of developing GAH and PE (8). Primiparae have a higher risk of developing hypertensive disorders than women with more than one previous gestation (8). Other conditions that appear to be risk factors for this gestational disorder are a history of chronic arterial hypertension and gestational hypertension in previous pregnancies. Patients who experience GAH during their first pregnancy may have a risk up to 7-fold higher of having GAH in subsequent pregnancies than women who do not have this disease (9).

Many Doppler investigations have been conducted in order to predict possible diseases that might affect the maternal-fetal binomial (10). Doppler ultrasound has a recognized role in the noninvasive assessment of uterine blood flow through the calculation of vascular impedance indices of the uterine arteries. A physiological reduction of uterine vascular resistance has already been demonstrated during a normal pregnancy (11).

The objective of the present study was to determine the influence of clinical and obstetrical history, as well as the history of body mass index, mean blood pressure, and of the indices of uterine artery impedance as associated factors contributing to the development of hypertension during pregnancy.

## Material and Methods

The present study was designed as a prospective cohort, with a protocol following the directives and norms of research on humans of the 196/96 Resolution of the National Health Council (Brazil). The study was approved by the Research Ethics Committee (protocol #4116/2008) of the University Hospital, Faculty of Medicine of Ribeirão Preto, University of São Paulo (HCFMRP-USP). The data for the study were obtained from the BRISA project (Brazilian Ribeirão Preto and São Luís Birth Cohort Studies, <https://nesca.fmrp.usp.br/brisa-botoes/>) entitled “Etiological factors of preterm birth and consequences of perinatal factors for the health of the child: birth cohorts from two Brazilian cities”.

A total of 1417 pregnant women were evaluated in a convenience sample due to the impossibility of obtaining a random sample representative of pregnant women from the population. Inclusion criteria were: gestational age of 20 to 25 weeks, a singleton fetus, and having been submitted to obstetrical ultrasound before 20 weeks for a more precise determination of gestational age. Exclusion criteria were: fetuses with congenital malformations or previously diagnosed chromosome syndromes, loss of follow-up between prenatal evaluation and childbirth, and participants with incomplete data. Patients that were not identified on the occasion of childbirth were also excluded. The patients who participated in the study were recruited at the basic health units of Ribeirão Preto by personal contact, telephone, or letter, with a visit being scheduled at the Outpatient Clinic of HCFMRP-USP.

The participants responded to a sociodemographic questionnaire regarding life habits, reproductive history, and previous diseases. Biometric parameters were evaluated and obstetrical ultrasound was performed. Maternal blood pressure was measured at two time points: before responding to the questionnaire and after the ultrasound exam, according to the norms of the VI Brazilian Directive of Hypertension (12), using a semiautomatic device. Before each measurement, the patients rested for 5 min. Blood pressure was measured with the patient in the sitting position, with uncrossed legs and feet resting on the floor, with the left arm placed at heart level and with the palm of the hand facing up. Systolic (SAP) and diastolic (DAP) arterial pressure values were recorded and mean arterial pressure (MAP) was later calculated as follows: SAP + 2 DAP/3. The height of the patients was measured with a wall anthropometer after the first blood pressure measurement. The patients were then weighed on a regularly calibrated floor digital scale. All data were annotated and digitized for the calculation of BMI as weight (kg)/height (m)^2^.

The ultrasound examinations with calculation of the pulsatility index and evaluation of the presence of protodiastolic notch were performed according to the 2009 criteria of Groom (13) as follows: the pregnant women were placed in horizontal decubitus with elevation of the headboard of 45 degrees, the transducer was placed on the lower quadrant of the abdomen with median angulation, the uterine artery was identified by color Doppler in the portion that crosses the external iliac artery superiorly, the sample volume was positioned at about 1 cm from this crossing, the flow rate curve was obtained with at least 5 similar waves of satisfactory quality, the impedance indices were measured, and the presence or absence of a diastolic notch was determined.

The exams were carried out using a Voluson 730 model Expert machine (General Electric Healthcare, Austria) and a Philips HDI 11 instrument (Philips, USA) using a two-dimensional convex probe at the frequency of 2–5 MHZ. The images were documented and filed on their own hard disks.

At the time of childbirth, 1306 patients of the initial cohort were identified by an active search of all maternities in the municipality. Eight hospitals in the city of Ribeirão Preto were visited daily. With the help of trained female interviewers, a new questionnaire was applied, this time regarding pregnancy and delivery data, as well as newborn data. Complementary data were obtained from the medical records. The final outcome was defined as the patient's report of arterial hypertension during the second half of pregnancy, defined as MAP of 140/90 mmHg or higher, diagnosed by a doctor or a nurse. All responses to the questionnaire were coded numerically with transformation and digitizing of all variables of the data bank. The data were reviewed in order to correct possible systematic errors. A data bank was created in MS-Access 2010 and the data were later compiled in a data base containing the two observation time points (pre- and post-natal) in order to permit statistical analysis of the variables to be tested.

### Statistical analysis

A descriptive analysis of the variables under study was first conducted, with the determination of means, ranges, and standard deviation. A binomial log regression model, multivariable analysis, was applied for the calculation of the risk attributed to the gestational hypertension outcome, followed by the calculation of the risk attributed to each variable with the adjustment of confounding factors, always with a 95% confidence interval (95%CI). All analyses were performed using the SAS software, version 9.2 (USA).

## Results

This study was conducted on 1306 pregnant women with a gestational age between 20 weeks (140 days) and 25 weeks and 6 days (181 days) (mean: 23.1 weeks, 161.8 days). Mean, minimum, maximum, and standard deviations of maternal and fetal data are presented in Table 1. Mean gestational age at childbirth was 273.4 days (39.0 weeks) and mean newborn weight was 3194 grams. The mean (0.89), minimum (0.32), and maximum (3.19) values and the standard deviation (0.29) of the impedance indices (calculated as the arithmetic mean of the values obtained for the two uterine arteries) are reported in Table 2. Qualitative analysis of the flow wave tracing with respect to the bilateral presence of a diastolic notch or to its unilateral presence or absence on both sides is presented in Table 2. Regarding personal history, 50 patients (3.65%) had a pre-gestational diagnosis of systemic arterial hypertension and 136 patients (9.90%) had a history of arterial hypertension during previous pregnancies. Post-childbirth analysis revealed 55.40% (759) vaginal deliveries, 40.30% (553) cesarean deliveries, and 4.20% (58) assisted vaginal deliveries (forceps or vacuum extractor). Of the patients interviewed, 190 (13.86%) responded that they had received a diagnosis of arterial hypertension from a doctor or a nurse during pregnancy.

**Table 1:** Description of the mother and fetus population studied at a gestational age of 20 to 25 weeks, and newborn data at birth.

	Mean	Minimum	Maximum	Standard Deviation
Maternal age (years)	25.8	12	45	6.06
Weight (kg)	70.5	40.1	165	14.7
BMI (kg/m^2^)	27.2	16.6	62.8	5.31
MAP (mmhg)	80.14	61.10	110	7.76
Parity (n)	2.07	1 (590)	18	1.34
GA (d)	161.8	140	181	1.34
EFW (g)	611	321	985	118.5
Baby's birth weight (g)	3191.3	376	4820	639.5
GA	273.4 d (39 w)	166 d (23.7 w)	306 d (43.7 w)	14.9

BMI: body mass index; MAP: mean arterial pressure; GA: gestational age in days (d) and weeks (w); EFW: estimated fetal weight.

**Table 2:** Impedance indices, rate of change in the pulsatility index, and presence of a diastolic notch in the flow rate wave of the uterine arteries.

	n (%)	Mean	Min	Max	SD
Impedance of the uterine arteries					
Pulsatility index		0.89	0.32	3.19	0.29
Resistance index		0.53	0.26	0.87	0.09
Systole-diastole ratio		2.29	1.21	8.6	0.73
Pulsatility index of the uterine arteries					
>p95	58 (4.39)				
<p95	1264 (95.6)				
Diastolic notch in the flow rate wave of the uterine arteries					
Absence of a notch	994 (75.2)				
Unilateral notch	227 (17.1)				
Bilateral notch	100 (7.6)				

SD: standard deviation; p95: 95th percentile of normality.

Table 3 shows the relative risk (RR) of the variables analyzed for the development of hypertension during pregnancy. The assessment of uterine artery pulsatilty index (PI) alone showed a relative risk of 2.69 when the value was above the 95th percentile for gestational age. The unilateral presence of a diastolic notch of the uterine arteries did not lead to an increased risk to develop hypertension, but its bilateral presence caused a significant 1.58 increase in risk. Women older than 35 years had a higher risk of arterial hypertension during pregnancy (RR: 2.82). Regarding parity, a history of 3 or more children caused a significant 1.68-fold increase in the risk of hypertension. A history of pre-gestational hypertension and of an increase in pressure levels during the first half of pregnancy also significantly increased the risk of gestational hypertension, with respective values of 5.35 and 5.78 times. A progressive increase in BMI was directly related to an increase in the RR of hypertension. BMI up to 24.9 involved an RR of 32, BMI of 25-29.9 involved an RR of 70, BMI of 30-34.9 an RR of 48, BMI of 35-39.9, an RR of 25, and BMI over 40 involved an RR of 14 (Table 3). MAP higher than 90 mmHg at the first visit also showed a strong correlation with the risk of gestational hypertension, increasing this risk 4-fold when MAP was 80.14 (SD: 7.76) (Table 1).

**Table 3:** Crude relative risk (RR) for the development of gestational hypertension.

	Gestational hypertension			
Variable	Yes	No	RR	95%CI
PI				
>p95	168 (88.89)	1080 (96.69)	1	1
<p95	21 (11.11)	37 (3.31)	2.69	1.86-3.89
Notch				
Absent	133 (70.37)	850 (76.10)	1	1
Unilateral	35 (18.56)	190 (17.01)	1.15	0.82-1.62
Bilateral	21 (11.11)	77 (6.89)	1.58	1.05-2.39
Maternal Age				
<19	14 (7.41)	120 (10.14)	1	1
19-34	145 (76.72)	925 (82.81)	1.30	0.77-2.18
≥35	30 (15.87)	72 (6.45)	2.82	1.58-5.03
Number of children				
0	77 (40.74)	493 (44.14)	1.02	0.75-1.36
1 or 2	77 (40.74)	502 (44.94)	1	1
3 or more	35 (18.52)	122 (10.92)	1.68	1.17-2.40
Previous AH				
Yes	33 (17.37)	17 (1.50)	5.35	4.18-6.85
No	157 (82.63)	1117 (98.50)	1	1
AH in the first half				
Yes	72 (38.30)	55 (4.91)	5.78	4.59-7.27
No	116 (61.70)	1066 (95.09)	1	1
BMI				
Up to 24.9	32 (16.93)	484 (43.33)	1	1
25-29.9	70 (37.04)	367 (32.86)	2.58	1.73-3.85
30-34.9	48 (25.43)	205 (18.35)	3.13	2.01-4.66
35-39.9	25 (13.23)	44 (3.94)	5.83	3.69-9.24
≥40	14 (7.4)	17 (1.52)	7.28	4.36-12.16
MAP				
≤90 mmHg	113 (59.78)	1026 (91.85)	1	1
≥90 mmHg	76 (40.21)	91 (8.14)	4.58	3.12-5.16

Data are reported as number and percent. PI: pulsatility index; AH: arterial hypertension; BMI: body mass index; MAP: mean arterial pressure; RR: relative risk; CI: confidence interval.

When we considered a combination of factors such as altered uterine artery PI and presence or absence of a diastolic notch in the flow rate waves in these arteries we observed an increase in RR for the development of arterial hypertension only in the presence of altered PI (0.89±0.29), regardless of the presence of a notch (Table 3). When multivariable analysis was performed for the calculation of RR specifically attributed to a risk factor, with exclusion of the deviations attributed to the variation in uterine artery PI, we observed that maternal age of more than 35 years, a maternal history of arterial hypertension, overweight, and obesity persisted as statistically significant risk factors, as shown in Table 4.

**Table 4:** Association of pulsatility index (PI) with the presence/absence of a diastolic notch and altered PI in the risk of developing arterial hypertension, and the relative risk adjusted to PI.

	Hypertension during pregnancy			
Notch/PI	Yes	No	RR	95%CI
Absent/Normal	127 (67.20)	841 (75.29)	1	1
Unilateral/Normal	29 (15.34)	174 (15.58)	1.09	0.75–1.58
Bilateral/Normal	12 (6.35)	65 (5.82)	1.19	0.69–2.05
Absent/Altered	6 (3.17)	9 (0.81)	3.05	1.61–5.79
Unilateral/Altered	6 (3.17)	16 (1.43)	2.08	1.03–4.19
Bilateral/Altered	9 (4.76)	12 (1.07)	3.27	1.94–5.49

Relative risk adjusted to PI	RR	95%CI
Age ≥35 years	2.07	1.02–4.20
History of SAH	2.15	1.40–3.30
AH during the first half of pregnancy	4.49	3.18–6.33
BMI between 25–29.9	1.89	1.23–2.90
BMI between 35–39.9	2.83	1.62–4.96
BMI above 40	3.24	1.67–6.30
Multiparity	1.13	0.75–1.71

This table presents the results of the binomial log model considering hypertension during pregnancy as an outcome and the variables age over 35 years, history of SAH, AH, and all ages from BMI to multiparity as covariates. RR: relative risk; CI: confidence interval, SAH: systemic arterial hypertension; AH: arterial hypertension; BMI: body mass index.

## Discussion

In the present study, we demonstrated that a history of pre-gestational systemic arterial hypertension and maternal age were strongly correlated with an increased risk to develop hypertension during pregnancy. BMI was related to a gradual increase of the risk to develop hypertension during pregnancy, with the risk being higher the higher the value of this index. MAP was found to be an important risk factor, as also was uterine artery PI, regardless of the presence or absence of a diastolic notch.

Morais et al. (14) determined the low weight, normal weight, overweight, and obesity curve according to gestational age (14) and observed that an appropriate BMI for a gestational age of 25 weeks would be 22.5 to 27 kg/m^2^. The mean BMI detected in the present study was 27.2 kg/m^2^, demonstrating a tendency towards obesity in the population under study.

Regarding maternal age, 10.27% of the deliveries occurred in patients younger than 19 years. According to the 2007 data of the Ministry of Health (15), this rate differs from the national proportion, which is 21.1%. The rate detected here in women older than 35 years was 7.85%, with values of 9.7 and 1.5% being recorded in Brazil as a whole and in the Southeast region, respectively (15). Mean parity was 2.07 gestations. Data of the Brazilian Institute of Geography and Statistics have shown a fecundity rate of 1.86 children per woman at the end of the fertile age (16).

The present study detected a 13.86% incidence of hypertension during pregnancy. A study conducted on 10,666 women detected a 9.6% rate of incidence of hypertensive syndromes of pregnancy (17). In a study conducted in Brazil, Assis et al. (17) detected a 14.5% incidence of hypertensive syndromes. Villar et al. (18), using data of the World Health Organization, evaluated 39,615 patients with a 7.0% rate of occurrence of gestational hypertension. In the same study, the authors mentioned obesity, a personal history of pre-eclampsia in a previous pregnancy, and advanced maternal age as risk factors, in agreement with the present study (19).

The present study confirmed literature data showing the importance of uterine artery PI as a predictor of hypertensive syndromes in pregnancy. Papageorghiou et al. (20) reported a significant increase in the incidence of PE in patients with PI above the 95th percentile, with a 6-fold higher relative risk (20). Another study detected high sensitivity for PE and intrauterine growth restriction (21) in the ultrasound evaluation of 8,202 pregnant women with a mean gestational age of 23 weeks.

A meta-analysis by Cnossen et al. (22) showed that the presence of a bilateral diastolic notch associated with increased PI in a Doppler study of the uterine arteries appears to be the best predictor of PE, with an RR of 7.5 times among low-risk patients (22). In the present study, there was a strong correlation between PI above the 95th percentile and hypertensive syndromes of pregnancy regardless of the presence or absence of a diastolic notch. The current study also demonstrated high correlation between obesity and increased risk of hypertensive syndromes during pregnancy. The increase was found to be higher the higher the BMI measured during the visit between 20 and 25 weeks. Another important point observed here was the high prevalence of overweight and obesity in the population under study, with the BMI of 27.0% of the pregnant women being higher than 30 kg/m^2^. This fact appears to be even more important because, among all risk factors evaluated in the present study, maternal BMI was the only one that could be changed by means of modification of life habits. Thus, maternal weight loss before pregnancy seems to be a practice that should be encouraged and directed during pre-conception visits of obese patients.

Advanced maternal age also increased the risk of hypertensive disorders, with a 2.82-fold increase in patients older than 35 years in the population studied. In a survey of more than 8 million births, Luke and Brown (23) detected an increased risk of developing hypertensive syndromes during pregnancy in primiparous patients aged 35-39 years, 40-44 years, and more than 45 years, with respective values of 1.13, 1.28, and 1.55 (23). In the present study, nulliparity did not increase the risk of developing hypertension during pregnancy, whereas a history of 3 or more children showed an increase of 1.68 times for this risk. Nulliparity is considered a risk factor for gestational hypertension (24). In a systematic review, Duckitt and Harrington (25) detected an almost 3-fold increase in the incidence of PE among nulliparous pregnant women.

A limitation of the study is that it was difficult to differentiate between gestational hypertension from chronic hypertension. We concluded that maternal age and a history of arterial hypertension were correlated with the risk to develop hypertension during pregnancy.
